# Favorite Prescriptions of Distinguished Practitioners, with Notes of Treatment

**Published:** 1889-01

**Authors:** 


### BOOK NOTICES AND REVIEWS.

### Favorite Prescriptions of Distinguished Practitioners, with notes on Treatment. Edited by B. W. Palmer, M. D., pages, 256, price, $2.75. New York: E. B. Treat & Co., 1888.

In this compilation we find prescriptions for almost any form of disease, and as they are recommended by celebrated physicians, they must be good! Can any one imagine a profession so silly as to use such prescriptions? Nothing could better illustrate the *science* of the old school than the demand for such works as this; patients are treated for *diseases* and in routine fashion. This method makes the practice of medicine easy enough for the physician, but it is rather rough on the patients.

